# Participant perspectives about decentralised trial procedures in a remote delivery nutrition intervention trial

**DOI:** 10.1017/jns.2025.10

**Published:** 2025-02-20

**Authors:** Naomi Davies, Rebecca F. Slykerman

**Affiliations:** Department of Psychological Medicine, The University of Auckland, Auckland, New Zealand

**Keywords:** Decentralised trials, Milk fat globule membrane, Nutrition intervention, Trial methods

## Abstract

Participant recruitment and retention are consistently recognised as significant, costly challenges in nutrition intervention trials. Decentralised study procedures address some of the recruitment and retention limitations in traditional trial methodology. Understanding participant perceptions and experiences of decentralised methods in nutrition studies is key to improving trial design and conduct. The aim of this study was to explore participant opinions about remote delivery of a dietary supplement intervention trial. Adults enrolled in a clinical trial of a milk fat globule membrane nutritional supplement for improvement of psychological wellbeing were invited to take part in a post-intervention interview. Interviews were conducted over video conferencing and transcribed. Using a semi-structured interview format six aspects of trial design were discussed: general processes, written instructions, contact throughout the study, self-collection of saliva samples, wearable device use, and cognitive assessment. Thematic analysis derived themes from the data for each of the aspects of trial conduct discussed. Seventy-three participants completed the interview. Interviewees reported a positive overall experience of the remote delivery procedures used. Accessible communication between researchers and participants and clear written instructions were identified as key to participant experience. Recall of instructions and adherence to the nutritional intervention was difficult for some respondents with suggestions made for facilitating this in future remote delivery nutrition studies. Use of wearables, in-home saliva sampling, and self-administered cognitive assessments were feasible and acceptable to most participants. The remote delivery of a nutritional intervention trial, including self-collected biological samples, is feasible and positively viewed by participants.

## Introduction

Nutrition intervention trials are fundamental to understanding the causal relationships between food supplements and health outcomes.^(1)^ Gold standard randomised controlled nutrition intervention studies play a pivotal role in determining the safety and effectiveness of supplements and dietary pattern interventions, ultimately shaping clinical practice and healthcare policies.^(2)^

Recruitment and retention of study participants are consistently recognised as significant and costly challenges within clinical trials.^(3–5)^ Traditional recruitment methods typically favour individuals with more flexible schedules who reside near the trial site. At the same time, minority groups, people in full-time employment and adults with caring responsibilities are underrepresented in clinical trial research. Studies necessitating in-person clinic visits present a further barrier to recruitment and retention. These factors can limit the validity of clinical trial results, making it difficult to extrapolate findings to a broader population.^(6)^

Decentralised clinical trials (DCTs) have emerged in the literature as an innovative solution to many of the limitations posed by traditional trial processes.^(7)^ Importantly, nutrition intervention studies are particularly well suited to remote delivery procedures because (i) they often investigate supplements or dietary patterns that people have ready access to and take as part of their general lifestyle, (ii) the instructions for use of nutritional supplements are not usually complex, (iii) and the outcomes assessed can often be ascertained by self-report measures or self-administered mechanisms.^(8)^ Decentralised methods include online eligibility checks and registration, phone, or online interviews, sending study materials via post, online cognitive assessments, and wearable devices linked to data collection platforms.^(9)^ These trial methodologies leverage technology to reduce the need for in-person visits to clinical sites while allowing participants to carry out study activities at a time and location that is convenient for them.

Decentralised procedures are utilised extensively in part or full for nutritional intervention and clinical trials.^(9–12)^ However, little is known about how participants experience these methods which is critical knowledge to improve trial design. A recent systematic analysis of the conduct of DCTs identified that despite heterogeneity in design and reporting, four broad themes of value, burden, safety and equity were evident.^(9)^ Within these themes, subthemes pertaining to reduced participant burden, maintaining privacy, and broadening access to the study were considered key advantages of DCTs, particularly for participant recruitment and retention. In contrast, increased burden on trial staff, lack of face-to-face interaction, and volume of trial activities were identified as potential disadvantages of DCTs. Participant views and experiences were underrepresented in this analysis, highlighting the need for participant centric feedback about DCTs.^(9)^ An exploration of stakeholder views used semi structured interviews with a sample of trial managers, principal investigators, and patient representatives, to canvas perceptions of DCT processes.^(13)^ Using clustering to generate specific interview themes, they identified that participant involvement and engagement were the most critical component to the success of DCTs where in-person contact is minimal. Factors influencing the enrolment and ongoing participation in remotely delivered dermatology studies have been investigated.^(8)^ A subgroup of participants in an Randomised Controlled Trial (RCT) of acne treatment took part in an interview about the remote methods adopted in the trial. Participants reported that sharing photos of their skin, carrying out consults via phone or video calls, and having the study medication and material delivered directly to their home helped the trial fit within their needs, added convenience and flexibility, and promoted a sense of feeling valued. The authors suggested that decentralised methods should be considered favourably when designing new trials and may enhance both recruitment and retention.^(8)^ These observations have helped identify some of the key successful components of remote trial processes, understanding participants’ needs and opinions on clinical trials is crucial and further research on how decentralised nutrition trials are experienced and perceived by participants is pertinent to maximise potential benefits and build an evidence base for remote trial delivery.^(14)^

We aimed to explore participants experience of remote delivery trial procedures in a dietary supplement intervention trial. More specifically, we aimed to investigate participant opinion about communication throughout the trial, remote self-administered cognitive assessment, collection of in-home saliva samples, and the utilisation of wearable fitness trackers.

## Methods

### Participants

Participants were adults aged 25 to 65 enrolled in the Employing Milk Phospholipids to Observe Wellbeing and Emotional Resilience (EMPOWER) study, a double blind, randomised trial to test Milk Fat Globule Membrane (MFGM) supplementation for improvement in psychological wellbeing in healthy adults. This fully decentralised study was designed to minimise participant burden through remote delivery of all trial procedures. The EMPOWER study aims, methodology and results have been described previously.^(15,16)^ In brief, participants were recruited between 31 January and 17 March 2022. Following online enrolment, and baseline demographic and questionnaire data collection, participants were randomly assigned to receive MFGM 600mg, MFGM 1200mg, or placebo at a ratio of 1:1:2. All consent and questionnaire data were collected and managed using a secure data collection platform. Participants were instructed to take the supplement once daily for 12 weeks. Participants completed psychological questionnaires and cognitive tests online at baseline and end of intervention. Self-collected saliva samples were obtained at baseline, mid-point (6 weeks) and end of intervention for analysis of cortisol levels. Sleep and activity data were collected via FitBit Charge 3 devices worn by participants for the duration of the trial.

### Data collection

At the completion of post-intervention data collection, all participants in the RCT were invited to take part in a qualitative sub-study interview about study processes. Interviews were conducted via Zoom video conferencing between 16 May and 15 June 2022. Audio recordings from Zoom were transcribed using Descript software (version 3).

Two interviewers conducted an approximately equal number of interviews. Training with the semi-structured questions and practice participants facilitated inter-interviewer consistency. One of the interviewers was involved in trial coordination and logistics and is an author of this manuscript. The second interviewer was involved only in interviewing and transcription but not in other aspects of the trial. Participants were asked about their experience of the study procedures. The interviews were guided by a semi-structured question format covering six broad domains including: General trial processes, written instructions, contact throughout the study, self-collected saliva process, wearable device use and cognitive assessments. Interviewers used the prompt “Can you explain further?” to invite participants to expand on answers. Interviews lasted 10-45 minutes, depending on how much the participant chose to elaborate on each question.

### Data analysis

Semi-structured interviews were transcribed by the two interviewers using Descript (version 100.00). The transcripts were analysed and interpreted using NVivo Qualitative Analysis software (version 14.23.0) by one of the interviewers and one separate researcher. A reflexive thematic analysis to identify themes emerging from the interview data was used due to its grounding in several key foundations, specifically, the awareness of the researchers subjectivity as well as the contextual understanding of the data.^(17)^ Participant comments were organised into the six aspects of trial procedures discussed during the interview. Themes emerging within each area and exemplar quotes from interview transcripts are provided to illustrate key themes.

## Results

### Participants

A total of 122 adults were enrolled into the EMPOWER study. Of the 106 participants who completed the end-of-intervention questionnaires, all were offered an end-of-study interview, and 73 participants completed an interview. Respondents and non-respondents to interview did not differ in intervention group (MFGM or placebo) (p = 0.43), gender (p = 0.55), or age (p = 0.25).

### Interview themes

Results are reported for each of the six domains discussed in interviews.

#### General trial processes

Of the 73 participants interviewed, 64 commented on the general trial procedures and how they felt about participating in the study. A theme of positive overall experience emerged from the data. Most participants found the study easy and enjoyable to participate in, and several said they thought the trial was well organised.

*“Easy. Yeah. Once I’ve got my head into the routine of taking the sachets, recording my Fitbit. Yeah, it was good. It didn’t impact on things. Very little, yeah. So, it was good.”*

*“It worked really well for a remote study for not seeing any subjects. It ran very well actually. I was wondering how it was going to go. Yeah. But it was good. I can see there was a lot of organizing going on.”*

The ability to participate in the trial despite not living close to a main city was reported as a positive aspect of the study.

*“I live in [a small town] so I can do it online. Yeah, yeah, yeah. For so many studies you have to live in a big center. And so that was quite appealing as well.”*

A few participants found the intervention period of 12 weeks long and had difficulty remembering daily study activities. There was a suggestion that a general study overview sheet to pin to the fridge would be helpful to remember the timeline and events of the study.

*“It was tedious, I guess, having to remember to do it every day for so long.”*

*“I thought that possibly at the beginning, there could have been one big overview sheet of all the steps you will need to go through”*

#### Provision of written instructions

Of the 73 participants interviewed, 60 people commented on the quality and usability of the instructions provided. There was a strong theme of a high quality of written communication, with participants reporting that they thought the instructions were of excellent quality, straightforward and easy to follow.

*“All the instructions and the details were very perfect.”*

*“Good. Easy to follow.”*

#### Amount of contact

All 73 participants interviewed commented on the amount of contact between researchers and participants during the trial. Most participants reported that the amount of communication from researchers was sufficient or suitable.

*“It was good. Not too much, but enough that the reminders were good. And yeah, it was, it was wonderful.”*

*“I guess more than enough? I never had to worry about who to contact if I had any questions.”*

A small number of participants felt that there could have been more contact and thought contact lacked a personal touch, referring to bulk SMS messages.

*“I think it could have been more. There were times when I thought, oh, it would’ve been interesting if they just sent out an email a day ahead to remind us of something the next day or something you did.”*

*“I sort of felt sometimes, I didn’t know if like I got those messages coming through saying to refresh, which I was doing constantly, so I don’t know if they were generic or aimed at me.”*

A prominent theme that emerged was the ability to quickly contact the study team, including timely responses, assistance with technology glitches, and feeling like there was always someone to contact if needed.

*“There’s always someone I could reach out to if I needed help with anything that was good.”*

*“I think you guys were actually quite onto it. Like I had questions as you know, and you guys were very responsive then on time. So I felt that was, I’ve never felt I was sort of like left out in the dark.”*

#### At home self-collected saliva sampling

Of the 73 participants interviewed, 62 people made comments pertaining to the self-collection saliva sampling process. The majority of participants found the process easy and straightforward, with no problem collecting and sending back the sample.

*“Actually, doing the saliva sample was relatively easy.”*

Some participants commented on the difficulty getting enough saliva in the morning, including having the ‘suck on the sides of their mouth’.

*“Incredibly difficult to generate such a small amount of saliva given how much it feels like you have in your mouth, but that was fine.”*

Some participants needed help to remember to collect the sample in the morning, stating they often had their morning coffee before they remembered and that they had to set an alarm on their phone to remind them.

*“That was the one thing I probably, you know, I wasn’t, yeah. I intended to do it most mornings and ate my breakfast and go, oh no, I’ve forgotten to do it.”*

#### Wearable fitness tracker use

Seventy participants commented on the wearable Fitbit wristwatch used in the trial. A theme of easy use and familiarity emerged where most participants made positive comments including on how easy the device was to use and found the process comfortable and familiar as they had already worn a fitness watch previously.

*“It was fine, actually.”*

*“I’ve been wearing Fitbit for like six or seven years. So I’m definitely used to it.”*

A theme of adjustment emerged from some participants, with some people taking time to adjust to wearing a wristwatch and others discussing difficulty in getting accustomed to wearing it during the night.

*“At first, I have to admit it had a strange feeling wearing it to bed, but then I got used to it.”*

*“Well, it is something that [was] maybe a little bit challenging.”*

*“It was fine. Except the last couple of weeks, I started having a reaction to the strap, and I got quite a rash.”*

*“I just feel like having sometimes the fact that having that watch on my wrist just could feel very constrained. Just feel not right.”*

#### Cognitive testing

Seventy two of the 73 participants interviewed provided feedback on their experience of in-home cognitive assessment using the computerised tests. Overall, most participants reported no issues with accessing the tests, the instructions of the testing and submitting the tests.

*“Perfect. No issues. The instructions on that were super clear.”*

A few participants commented that they thought the instructions were tedious to listen to (especially the second time around), they had browser incompatibility, and that they had technical issues.

*“The cognitive tests had very detailed, slow instructions. And sometimes, I was getting a little bit frustrated with how slow they were when I knew what to do, especially the second time.”*

*“It didn’t work on Linux. Yeah. I had to use a windows machine.”*

*“When I had to [submit] send the test results back, I have to do it a few times. And then it just didn’t go through.”*

*“… there were a lot of technical issues. it froze a bunch of times.”*

## Discussion

We present the findings from a qualitative investigation of participant experience captured in interviews with healthy adults who took part in a DCT of MFGM supplementation for psychological health.^(15)^ The results extend current understanding of the opinions of participants about decentralised methods in a dietary supplement intervention trial. A general theme of positivity about decentralised methods was evident in the data. A key motivator for initial enrolment and ongoing engagement in the trial was the feeling of inclusion by participants who were not based in main cities, and surprising ease of participation in remotely delivered procedures. These positive themes are consistent with previous reports that indicate remote trial delivery facilitates registration and participation due to minimal interference with existing commitments.^(8,18,19)^

Communication was identified as an important aspect of trial conduct. Participants reported that high quality written instructions provided for the different components of the study facilitated engagement. Clear and succinct written instructions have been identified as paramount for robust data collection in nutrition trials,^(20)^ particularly when communicating with participants of diverse comprehension and sociocultural backgrounds.^(21)^ In studies utilising completely decentralised methods, straightforward and easy to follow instructions for sample collection and study processes that avoid jargon are especially important to ensure that information is accurately communicated to participants to maximise adherence to study procedures.

Participants were positive about the amount of contact received from the research team, which included weekly SMS reminders to charge the wearable device, fortnightly reminders to report any adverse events, and reminders to complete the online questions 5 and 7 days after receiving the original link via SMS and email. Participants were also positive about the ability to contact the researchers and receive a prompt response. However, some participants reported feeling that messages lacked a personal touch, and that they were unsure if the messages they received were generic or individualised. This is in line with some of the disadvantages of DCTs previously reported, where participants commented that remote delivery of study assessments felt impersonal and that they missed the time to chat with study staff and see their body language.^(22)^ Personal, and bi-directional communication throughout a DCT is essential, and ease of communication that can be delivered and received in both directions between participants and researchers facilitates engagement and retention. Underpinning this communication is the building of trust between the participant and research team. When researchers provide timely, high-quality, communication this assists to build a relationship with participants. An analysis of psychosocial factors influencing trial participation found that trust in the research and research team, and ease of participation emerged as the primary facilitators that prompt members of the public to participate in clinical trials.^(23)^

An overall theme emerged from interview data about the need to remember to complete different study requirements, which is pertinent when considering a *daily* nutrition intervention. Multiple components of the study required participants to remember to execute tasks at specific time points including, self-collecting and returning saliva samples, syncing Fitbit watches, and taking the daily supplement. Earlier studies have reported reduced adherence to a daily task in light of dynamic external life influences.^(24,25)^ Limiting protocol complexity to only those procedures required to address the scientific questions of the study has been identified as a recommendation for improving clinical trial design.^(14)^ Balancing participant burden with the potential convenience offered by in-home self-collection tasks is a particular consideration for decentralised trials.^(13)^ Participants in our study suggested that researchers should provide a single study overview sheet to help guide and remind the participants about what to expect and the study timeline. This could be incorporated into future trial design.

In nutritional intervention trials, the goal is always to maximise the generalisability of the findings while producing the highest quality data and level of scientific integrity, all whilst balancing adherence and participant burden.^(26)^ The length of the intervention should be determined by the anticipated timeframe required for the outcomes of interest to attain a biologically significant change.^(26)^ A small number of participants found repeated study activities including taking the daily supplement and syncing the Fitbit wristwatch became tedious. However, of the 122 participants enrolled in the EMPOWER trial, 89.3% and 86.9% of participants completed the midpoint and end-point assessments, respectively, demonstrating that the trial demands were feasible and acceptable to most participants.

Our dietary supplement trial included participant self-collection of saliva samples which were returned to the research team by courier. Participants reported no difficulties with saliva collection and return. Saliva collection in-clinic offers the benefit of a trained person overseeing the collection and removes the risk of the samples being lost or damaged during postal return. Conversely, biological sample collection is more patient-centric if it is less invasive and can be collected remotely.^(27)^ We did not encounter difficulties with lost samples, and only recorded a single instance of a sample being damaged in the post in our study, leading us to conclude that the benefits of reduced participant burden of in-home saliva collection far outweigh the potential limitations.

The additional components of our trial, specifically the use of a wearable device and online cognitive assessment, were acceptable to participants. Wearable fitness tracker devices are popular worldwide, and this was evident in our cohort, where a third of participants reported having no issue wearing the device and adapting to it as they usually wore one anyway (of either the same or different brands). Wearable devices offer a practical and low-cost tool to monitor and address physical activity in nutritional intervention trials,^(28)^ and given their popularity, they are a feasible method for data collection for decentralised trials. For remote cognitive testing, interview data from this study suggests that participants found the tests clear and straightforward with few technical issues. Overall, although the breadth of in person administered cognitive tests is more extensive,^(29)^ and there are fewer validated online self-administered tests, we have demonstrated the feasibility of remote cognitive testing in a decentralised trial. It is important to note that our study was in adults, and older adults or those less familiar with the use of computers in their everyday lives may find remote cognitive assessment procedures more difficult without the assistance of an in-person researcher. However, this does not appear to impact the participants performance on the tests.^(30)^

Our results may have been influenced by self-selection bias. It is possible that participants who took part in the interviews did so because they were interested in research activities and had a positive experience of the trial including the decentralised design. Participants who needed help with aspects of remote delivery procedures may not have continued in the study and therefore our results may not reflect their experience. Interview respondents and non-respondents did not differ in intervention group (MFGM or placebo), age, or gender, and our results are therefore more likely to represent the overall group of trial participants who completed end of intervention quantitative data collection. Social desirability bias^(31)^ can influence interviews where participants may have responded positively to questions about trial procedures because they unconsciously want to please the interviewer. This may have led to underrepresentation of opinions about barriers to trial participation. However, our results are consistent with those of previous studies reporting favourable participant experiences in decentralised trials.^(8)^ Furthermore, participants offered feedback about ways in which their experience could have been improved suggesting that at least some participants were comfortable sharing these views. The large sample size of this qualitative investigation of participant experience is a strength of the research and is likely to have captured a range of participant opinions.

Researchers play a key role in the analysis process; their unique backgrounds and experiences may shape the identification of themes in the qualitative data. A reflexive approach prompts authors to critically assess how their viewpoints influence the thematic outcomes and ensure a more nuanced understanding of the data.^(32)^ We acknowledge that one of the interviewers was involved in the study design and logistics, which may have influenced how the participants’ answers were perceived and the definitions of emerging themes. For this reason, another interviewer conducted and transcribed interviews and a second researcher was involved in analysis and data interpretation.

### Summary and conclusion

General recommendations for decentralised approaches that can be extrapolated from this work include: 1) the importance of clear and precise written instructions for any self-administered study activity including biological sample collection, 2) providing an overall study timeline at the commencement of the study to guide participants through upcoming study procedures, and 3) Ensuring the availability of researchers to promptly reply to any queries or correspondence from the study participants via multiple modes such as SMS, email, and phone.

Remotely delivered trial procedures in randomised clinical trials of nutritional supplements are feasible and positively experienced by participants. Decentralised trials present the advantage of reducing barriers to participation. Careful consideration of the participant experience can facilitate successful recruitment, retention, and adherence. These findings offer insights into the views of participants and their relevance to decentralised trials. Future studies could include assessments of participant experience to further inform the practical design of decentralised trial methods.

## Supporting information

Davies and Slykerman supplementary material 1 Davies and Slykerman supplementary material

Davies and Slykerman supplementary material 2 Davies and Slykerman supplementary material

## Supplementary material

The supplementary material for this article can be found at https://doi.org/10.1017/jns.2025.10
