# Supplementary material for: Participant perspectives about decentralised trial procedures in a remote delivery nutrition intervention trial
Source: J Nutr Sci. 2025 Feb 20;14:e13. doi: 10.1017/jns.2025.10 (PMC11894400; doi:10.1017/jns.2025.10)
Supplement: Davies and Slykerman supplementary material 2 — Davies and Slykerman supplementary material [file S2048679025000102sup002.docx]

**EMPOWER Study: Qualitative Interview Questions**

Firstly, thank you for taking part in our study, we are really appreciative of the time and effort you have contributed to this research. This interview is an opportunity for you to talk about your experience and give us any feedback you would like to.

This interview will consist of a set of formal questions, and at the end you will have a chance to add anything else you think we have missed.

If it is ok with you, I would like to record the sound of this interview for my note taking.

-----------------------------------------------------[Once recording]----------------------------------------------------------

Thanks for confirming that I can record this interview.

The first set of questions are around your participation in the study:

**Part 1:**

1. Firstly, what appealed to you about this study?
2. How did you find taking part in the study overall?
3. Did you feel like we made enough contact with you throughout the study?
4. How did you find taking the supplement?
5. How did you take the powder?
6. How many foil packets do you have left? [Ideally the person would count them and give an exact number]
   1. How many would you estimate you have left? [If the participant is unsure, offer some bands e.g. <10,10-15, 15020]

[IF NUMBER IS NOT KNOWN:]

- 1. In the last week you were taking the powder how many days out of seven did you take it? Was this typical of the number of times generally you took it each week?

1. How did you find collecting the saliva sample?
2. How was it wearing the Fitbit for 14 weeks?
3. How did you find the instructions for the:
   1. Supplement
   2. Fitbit
   3. Saliva samples
   4. Cognitive tests
4. Did you have any computer issues with the cognition tests?
5. Do you have any other comments around the technical bits of the study?

**Part 2:**

1. Can you tell me a bit about your general wellbeing throughout the course of the study?
2. Did you notice any improvements in your physical or mental wellbeing during the study?

If so, can you describe them?

1. Do you think you had any side effects when you started taking the powder?

If yes, did they resolve or were they persist?

1. Have you found yourself more or less physically active since starting this study?
2. Did you notice any difference in your sleep during the study?
3. Did you notice any changes in your physical energy during the study?
4. Did you notice any difference in your mental energy – for example, your ability to focus and stick with tasks?
5. Did you notice any difference in mood throughout the study?
6. Which group do you think you were in?
7. Did you have covid-19 during this study? If so, do you remember when?
8. And finally, do you have any other comments you would like to add regarding this study?
